# Genetic, Epigenetic, and Metabolic Maintenance of Neural Stem Cells During Aging

**DOI:** 10.1093/geroni/igaa057.2664

**Published:** 2020-12-16

**Authors:** Ashley Webb

**Affiliations:** Brown University, Providence, Rhode Island, United States

## Abstract

Tight metabolic regulation is essential to maintain stem cell homeostasis and support healthy aging. With age, metabolic alterations cause neural stem cell (NSC) dysfunction and are associated with a decline in neurogenesis, but the underlying mechanisms are not known. Aged stem cells display defects in the autophagy-lysosomal pathway that may disrupt mitochondrial dynamics, resulting in metabolic disruptions that alter self-renewal and differentiation potential. We have used genomic and functional approaches to investigate the metabolic mechanisms that support NSCs throughout aging. We found that mitochondrial and mitophagy gene networks as well as mitophagy dynamics are differentially regulated between the quiescent and activated states and become dysregulated with age. This work provides new insight into the metabolic regulation of NSCs and may lead to strategies to enhance neurogenesis in the context of aging and neurodegenerative disease.

